# Acute Lymphoblastic Leukemia Presenting With Severe Acute Kidney Injury Requiring Hemodialysis due to Kidney Infiltration: A Case Report

**DOI:** 10.1002/ccr3.73111

**Published:** 2026-07-06

**Authors:** Semin Cho, Soyean Kwon, Jeong Hwan Park, Jin Hyun Park, Ki Hwan Kim, Jin Soo Kim, Eun Youn Roh, In Sil Choi, Ji Eun Kim, Song Yi Park

**Affiliations:** ^1^ Department of Internal Medicine Chung‐Ang University Gwangmyeong Hospital Gyeongi Republic of Korea; ^2^ Department of Internal Medicine Seoul Metropolitan Government Seoul National University Boramae Medical Center Seoul Republic of Korea; ^3^ Department of Pathology Seoul Metropolitan Government Seoul National University Boramae Medical Center Seoul Republic of Korea; ^4^ Department of Laboratory Medicine Seoul Metropolitan Government Seoul National University Boramae Medical Center Seoul Republic of Korea

**Keywords:** acute kidney injury, acute lymphoblastic leukemia, aleukemic presentation, hemodialysis, kidney biopsy, leukemic infiltration

## Abstract

A 66‐year‐old man presented with dialysis‐dependent acute kidney injury without cytopenia or peripheral blasts. Kidney biopsy revealed leukemic infiltration and led to the diagnosis of Philadelphia chromosome‐negative B‐acute lymphoblastic leukemia. Chemotherapy resulted in remission and recovery of renal function, highlighting the importance of early biopsy in unexplained acute kidney injury.

## Key Clinical Message

Direct leukemic infiltration of the kidney is a rare cause of severe acute kidney injury in adults with acute lymphoblastic leukemia. Early recognition through kidney biopsy and prompt chemotherapy may allow recovery of kidney function and achieve hematologic remission.

## 1 Introduction

Acute lymphoblastic leukemia (ALL) is a malignant hematologic disorder characterized by the uncontrolled proliferation of immature lymphoid progenitor cells. These abnormal cells infiltrate the bone marrow and extramedullary tissues such as the spleen, liver, lymph nodes, and bones, resulting in marrow failure and systemic symptoms. Clinically, patients often present with anemia, thrombocytopenia, neutropenia, fever, fatigue, bone pain, and lymphadenopathy. The disease arises from multistep somatic mutations that disrupt cellular processes including proliferation, differentiation, and apoptosis, although the exact etiology remains largely unknown [1]. Most clinical signs of ALL result from malignant lymphoid cell accumulation, presenting with symptoms like fever, weight loss, bleeding, fatigue, infection, lymphadenopathy, organomegaly, and sometimes CNS or mediastinal masses [2]. More commonly, kidney failure in hematolymphoid malignancies results from factors such as dehydration, hypercalcemia, ureteral obstruction, vascular compromise, paraproteinemia, glomerulonephritis, or treatment‐related side effects like tumor lysis syndrome [3]. Direct kidney involvement in leukemia can be a cause of acute kidney injury (AKI); however, tissue biopsy is rarely performed because the diagnosis is often established through bone marrow examination. Although cases of ALL presenting with AKI due to leukemic renal infiltration have been reported in both pediatric and adult patients [4, 5, 6], dialysis‐dependent AKI as the initial manifestation of adult ALL remains exceedingly rare. Therefore, we report a case of Philadelphia chromosome‐negative B‐ALL presenting with severe AKI requiring hemodialysis due to biopsy‐proven renal leukemic infiltration.

## 2 Case History/Examination

A 66‐year‐old Asian man presented with a 1 week history of abdominal pain. Initial laboratory evaluation revealed severe acute kidney injury with blood urea nitrogen (BUN) of 83 mg/dL, serum creatinine of 9.86 mg/dL, and an estimated glomerular filtration rate (eGFR) of 5 mL/min/1.73 m^2^, calculated using the chronic kidney disease epidemiology collaboration (CKD‐EPI) equation. The patient had a 15 year history of hypertension and dyslipidemia, and had been receiving candesartan, amlodipine, and rosuvastatin. A routine health examination performed a month prior to presentation demonstrated normal kidney function. He denied any family history of kidney disease, as well as smoking or alcohol consumption. Notably, the patient reported a lifelong tendency to bruise easily.

On physical examination, the abdomen was soft with normal bowel sounds, and there was no peripheral edema. The patient had oliguria and symptoms suggestive of uremia, including nausea and vomiting, and was admitted for further management. Hemodialysis was initiated via a tunneled catheter. Additional laboratory findings showed serum potassium of 6.4 mmol/L, serum phosphorus of 6.5 mg/dL, serum calcium of 9.3 mg/dL, serum uric acid of 24.7 mg/dL, and serum total carbon dioxide (tCO_2_) of 21.0 mmol/L. Liver function tests revealed aspartate aminotransferase (AST) of 84 U/L, alanine aminotransferase (ALT) of 62 U/L, alkaline phosphatase (ALP) of 228 U/L, gamma‐glutamyl transferase (GGT) of 239 U/L, and lactate dehydrogenase (LDH) of 1898 U/L. Interestingly, complete blood count (CBC) demonstrated white blood cell (WBC) of 11.96 × 10^3^/μL, hemoglobin (Hb) of 12.1 g/dL, platelet of 127 × 10^3^/μL, and atypical lymphocytes of 11%, without evidence of blasts or immature cells in the peripheral blood. Immunologic tests performed to differentiate glomerulonephritis, including anti‐nuclear antibody, myeloperoxidase (MPO)/proteinase 3 (PR3) antibody, and rheumatoid factor (RF), all yielded negative results. Serum immunoglobulins were immunoglobulin G (IgG) of 839 mg/dL, immunoglobulin A (IgA) of 72 mg/dL, and immunoglobulin M (IgM) of 29 mg/dL. The computed tomography (CT) scan performed to evaluate the etiology demonstrated diffuse swelling of both kidneys with decreased enhancement (Figure 1A), along with retroperitoneal edema and fascial thickening (Figure 1B).

**FIGURE 1:**
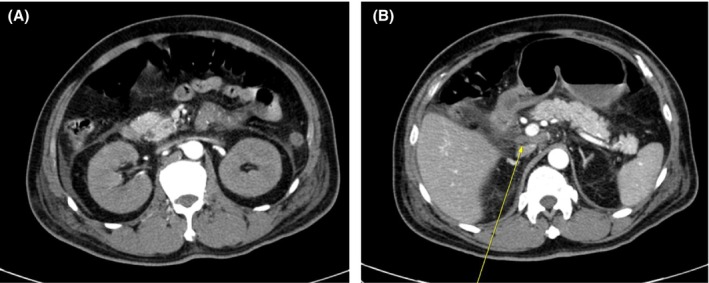
Computed tomography of the abdomen and pelvis with contrast. (A) Diffuse swelling of both kidneys with decreased enhancement. (B) Retroperitoneal edema and fascial thickening without pancreatic swelling. The yellow arrow indicates multiple enlarged periportal lymph nodes.

## 3 Differential Diagnosis, Investigations and Treatment

Given the unexplained severe AKI accompanied by bilateral nephromegaly, markedly elevated LDH and uric acid levels, and atypical lymphocytes in the peripheral blood, both bone marrow examination and kidney biopsy were pursued as part of the diagnostic workup for a possible hematologic malignancy and to determine the cause of kidney dysfunction.

Bone marrow aspiration and biopsy were performed to evaluate for lymphoid malignancy. The marrow was markedly hypercellular (90%–100%) with extensive replacement by blasts. Differential counts revealed 85.5% lymphoblasts, and trilineage hematopoiesis was markedly suppressed (Figure 2A). Immunohistochemical staining showed that the blasts were negative for CD3 (Figure 2B), faintly positive for CD79a (Figure 2C), and strongly positive for PAX5 (Figure 2D) and terminal deoxynucleotidyl transferase (TdT) (Figure 2E), supporting the diagnosis of B‐ALL. Additionally, fluorescence in situ hybridization (FISH) and polymerase chain reaction (PCR) tests for the *BCR/ABL1* fusion were both negative, allowing the diagnosis of Philadelphia chromosome‐negative B‐ALL.

**FIGURE 2:**
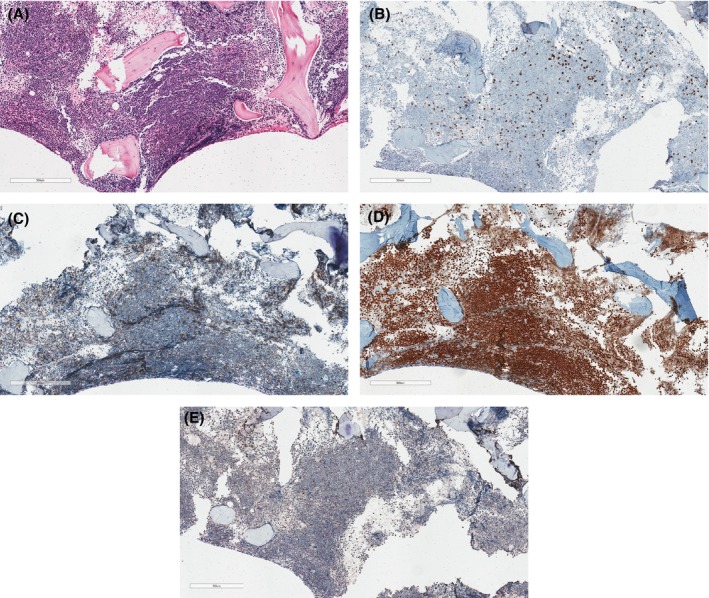
Bone marrow examination findings support a diagnosis of B‐lymphoblastic leukemia/lymphoma. Atypical lymphoid cells are diffusely replacing the marrow cavity (hematoxylin eosin H&E) (A). The cells are negative for CD3 (B), faintly positive for CD79a (C), and uniformly positive for PAX5 (D) and terminal deoxynucleotidyl transferase (TdT, E). All images at magnification ×100.

Kidney biopsy, performed as part of the concurrent diagnostic evaluation, revealed diffuse infiltration by atypical lymphoid cells. Light microscopy demonstrated 10 glomeruli, with 40% global sclerosis and mild mesangial hypercellularity. Tubulointerstitial findings included focal tubular atrophy, mild interstitial fibrosis, and diffuse infiltration by atypical lymphoid cells. Immunofluorescence was negative for immunoglobulin and complement deposition. Electron microscopy showed no immune‐type electron‐dense deposits, with only focal glomerular basement membrane thinning and mild foot process effacement. Immunohistochemical staining demonstrated *PAX5* positivity in infiltrating lymphoid cells, consistent with precursor B‐cell lineage. Light microscopy demonstrated diffuse infiltration of atypical lymphoid cells (Figure 3A). The infiltrating cells were negative for CD3 (Figure 3B), showed focal positivity for CD20 (Figure 3C) and TdT (Figure 3D), and demonstrated strong diffuse positivity for PAX5 (Figure 3E), supporting leukemic infiltration of the kidney.

**FIGURE 3:**
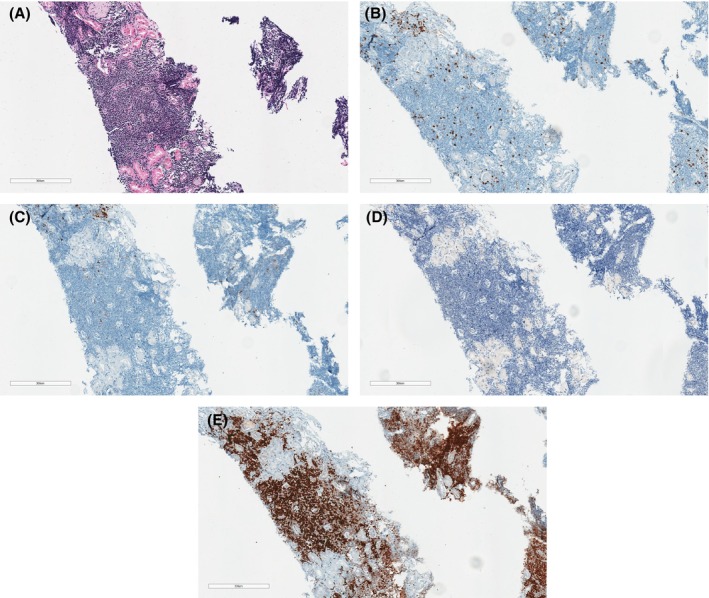
Kidney needle biopsy findings suggestive of involvement from hematolymphoid malignancy. A. Atypical, monotonous lymphoid cells are diffusely infiltrating the kidney parenchyma (H&E) (A), B–E. Immunohistochemical staining of the kidney biopsy. Tumor cells are negative for CD3 (B), show only focal/scant positivity for CD20 (C) and TdT (D), and demonstrate strong diffuse positivity for PAX5 (E).

Based on these findings, the patient was diagnosed with Philadelphia chromosome‐negative B‐ALL with direct kidney involvement presenting as AKI requiring hemodialysis. While the patient was undergoing hemodialysis, induction chemotherapy for B‐ALL was initiated, consisting of daunorubicin, vincristine, methylprednisolone, L‐asparaginase, and intrathecal methotrexate (MTX). These medications were administered with doses adjusted for kidney function.

## 4 Conclusion and Results

The patient tolerated the chemotherapy well, with no significant side effects observed. Improvement in kidney function was observed 2 weeks after the initiation of chemotherapy, leading to a gradual reduction in hemodialysis sessions. One month after initiation of therapy, he was able to discontinue hemodialysis entirely. Following discontinuation, laboratory tests showed a BUN level of 22 mg/dL, a serum creatinine level of 2.60 mg/dL, and an estimated glomerular filtration rate of 24.8 mL/min/1,73 m^2^. Urine output was maintained at more than 1000 mL per day. Moreover, follow‐up bone marrow examination after induction therapy demonstrated morphologic remission, and minimal residual disease (MRD) testing was negative, indicating that he had achieved complete remission. Subsequently, the patient received consolidation therapy, and his kidney function has been gradually improving without the need for kidney replacement therapy.

In conclusion, this case highlights that direct leukemic infiltration should be considered as a rare but important cause of AKI in patients with ALL. Early recognition through kidney biopsy can enable timely initiation of appropriate chemotherapy, leading to both hematologic remission and kidney recovery. Awareness of this potential presentation may help clinicians avoid delays in diagnosis and optimize patient outcomes.

## 5 Discussion

ALL is a malignant hematologic disorder characterized by the clonal proliferation of immature lymphoid precursors infiltrating the bone marrow and various extramedullary sites. This process results in marrow failure and diverse systemic manifestations, including anemia, thrombocytopenia, and lymphadenopathy [1]. The kidney is a common extranodal site of involvement in hematolymphoid malignancies. However, clinically significant kidney dysfunction caused by diffuse leukemic or lymphomatous infiltration is rare. In most cases, kidney impairment in such patients results from secondary causes, including tumor lysis syndrome, dehydration, hypercalcemia, ureteral obstruction, vascular compromise, or drug‐induced nephrotoxicity, rather than direct parenchymal infiltration [3].

Although kidney infiltration by hematologic malignancies is frequently observed in autopsy studies, clinically apparent kidney dysfunction resulting from such infiltration is exceedingly rare. Autopsy data have demonstrated kidney involvement in up to 60%–90% of patients with leukemia or lymphoma, with reported infiltration in approximately 54% of ALL cases. Nevertheless, kidney failure directly attributable to leukemic or lymphomatous infiltration occurs in only about 1% of patients with acute leukemia. These findings indicate that, despite the high prevalence of subclinical kidney involvement, clinically significant AKI caused by direct leukemic infiltration remains exceptionally uncommon, underscoring the rarity and diagnostic challenge of cases such as the present one [7].

Several adult cases of ALL presenting with AKI secondary to leukemic renal infiltration have previously been reported [5, 6]. In many of these reports, patients presented with bilateral nephromegaly and impaired kidney function, and the diagnosis was established through kidney biopsy in conjunction with hematologic evaluation. However, adult cases presenting with dialysis‐dependent AKI remain particularly uncommon. In the present case, severe AKI requiring hemodialysis was the initial manifestation of ALL, and prompt induction chemotherapy resulted in both hematologic remission and successful discontinuation of dialysis.

In the present case, a 66‐year‐old man presented with severe AKI requiring hemodialysis. Initial laboratory findings revealed markedly elevated serum creatinine and uric acid levels, while the CBC showed no cytopenia and only 11% atypical lymphocytes without blasts or immature cells in the peripheral blood, suggesting an aleukemic presentation. Kidney biopsy was subsequently performed and demonstrated diffuse infiltration of lymphoblasts positive for B‐lineage markers, confirming the diagnosis of B‐ALL with kidney involvement.

The aleukemic presentation of this case is particularly noteworthy. Unlike the typical presentation of ALL, which is often accompanied by peripheral blood abnormalities reflecting bone marrow involvement, our patient presented primarily with severe AKI requiring hemodialysis, while peripheral blood examination showed neither blasts nor clinically significant cytopenias. Such an atypical presentation may delay consideration of an underlying hematologic malignancy and increase the risk of diagnostic delay. Therefore, in patients with unexplained AKI accompanied by bilateral nephromegaly, elevated LDH levels, or atypical lymphoid cells in the peripheral blood, clinicians should maintain a high index of suspicion for leukemic infiltration even in the absence of classic hematologic findings.

In contrast to secondary causes of AKI commonly observed in hematologic malignancies, such as tumor lysis syndrome or drug‐induced nephrotoxicity, the kidney dysfunction in this patient was attributed to direct leukemic infiltration. Had AKI been secondary in nature, chemotherapy initiation might have been delayed due to concerns regarding kidney impairment. However, recognizing that the kidney failure resulted from leukemic involvement, we considered that rapid cytoreduction could facilitate kidney recovery. Consequently, chemotherapy was initiated promptly with appropriate dose adjustment according to kidney function, leading to both complete remission of leukemia and restoration of kidney function. The temporal relationship between induction chemotherapy and renal recovery further supports leukemic infiltration as the primary cause of AKI. Following treatment initiation, serum creatinine levels progressively declined, and the patient was ultimately able to discontinue hemodialysis with sustained improvement in kidney function (Figure 4).

**FIGURE 4:**
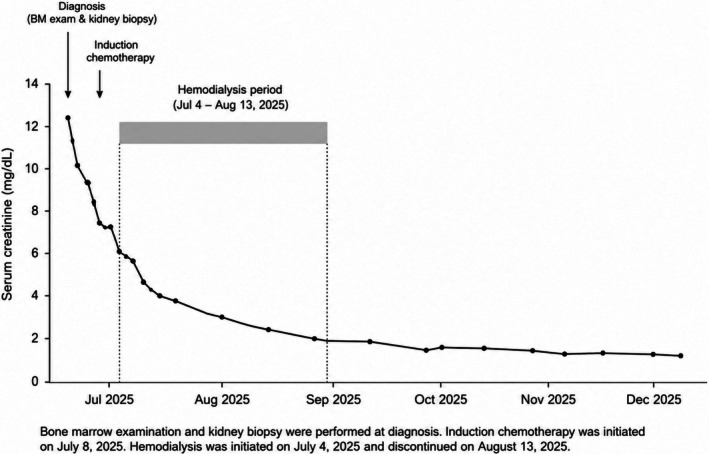
Clinical course of renal function during treatment. Serial serum creatinine levels are shown in relation to major clinical events, including bone marrow examination, kidney biopsy, initiation of induction chemotherapy, and the period of hemodialysis. Renal function progressively improved following induction chemotherapy, allowing successful discontinuation of hemodialysis on Day 40 after presentation.

Given these findings, this case underscores the importance of considering direct leukemic infiltration as a potential etiology of AKI in patients with hematologic malignancies. Because secondary causes such as tumor lysis syndrome or drug‐induced nephrotoxicity are far more common, the possibility of leukemic kidney involvement may easily be overlooked, especially when peripheral blasts are absent or cytopenia is not evident. Therefore, when the clinical picture is atypical or the cause of kidney dysfunction remains unclear, early and active evaluation with kidney biopsy should be strongly considered. Timely identification of leukemic infiltration can facilitate prompt initiation of appropriate chemotherapy, which not only addresses the underlying malignancy but may also lead to rapid kidney recovery and improved overall outcomes.

## 6 Patient Perspective

The patient reported that receiving the diagnosis and undergoing intensive induction chemotherapy was initially overwhelming and physically challenging. However, after achieving complete remission following induction treatment, the patient expressed a strong sense of relief and optimism. At the time of writing, the patient is undergoing consolidation therapy and reported overall satisfaction with the treatment course and current clinical condition, expressing confidence in the ongoing management plan.

## Author Contributions

**Jeong Hwan Park:** resources, methodology, visualization. **Soyean Kwon:** data curation, validation. **In Sil Choi:** supervision. **Semin Cho:** conceptualization, investigation, methodology, writing – review and editing, writing – original draft. **Jin Soo Kim:** supervision. **Eun Youn Roh:** methodology, visualization, resources. **Ki Hwan Kim:** supervision. **Ji Eun Kim:** data curation, visualization, writing – original draft, resources. **Song Yi Park:** conceptualization, writing – original draft, writing – review and editing. **Jin Hyun Park:** supervision.

## Funding

The authors have nothing to report.

## Ethics Statement

This study protocol was reviewed and approved by the institutional review board of Seoul Metropolitan Government Seoul National University Boramae Medical Center [20‐2025‐71]. Written informed consent was obtained from the patient for participation in this study.

## Consent

Written informed consent was obtained from the patient for publication of this case report and any accompanying images. A copy of the written consent is available for review by the Editor‐in‐Chief of this journal upon request.

## Conflicts of Interest

The authors declare no conflicts of interest.

## Data Availability

All data generated or analyzed during this study are included in this published article. Additional data are available from the corresponding author upon reasonable request.
